# Elevated plasma aldosterone-to-renin ratio as a potential risk marker of adverse left ventricular remodeling: a cross-sectional study

**DOI:** 10.3389/fmed.2025.1703635

**Published:** 2026-01-07

**Authors:** Mingjie Xu, Yushuang Wei, Mingli Li, Zengnan Mo, Boteng Yan

**Affiliations:** 1 Department of Urology, Hunan University of Medicine General Hospital, Huaihua, Hunan, China; 2 Center for Genomic and Personalized Medicine, Guangxi key Laboratory for Genomic and Personalized Medicine, Guangxi Collaborative Innovation Center for Genomic and Personalized Medicine, Guangxi Medical University, Nanning, Guangxi, China; 3 School of Public Health, Guangxi Medical University, Nanning, Guangxi, China; 4 Institute of Urology and Nephrology, First Affiliated Hospital of Guangxi Medical University, Guangxi Medical University, Nanning, Guangxi, China

**Keywords:** aldosterone-to-renin ratio, adverse left ventricular remodeling, left ventricular, aldosterone, left ventricular hypertrophy

## Abstract

**Background:**

Recent evidence has suggested that primary aldosteronism (PA) is the predominant cause of secondary hypertension and is linked to adverse left ventricular (LV) remodeling. However, few studies have investigated the potential associations of aldosterone-to-renin ratio (ARR), an important parameter for PA screening, with the risk of adverse LV remodeling in the Chinese population. This study aimed to investigate the associations of ARR, plasma aldosterone concentration (PAC), and plasma renin concentration (PRC) with adverse LV remodeling in a population from Guangxi, China.

**Methods:**

The analyzed data were primarily obtained from the First Affiliated Hospital of Guangxi Medical University and the First People’s Hospital of Yulin City during the period from September 2022 to March 2024. A total of 724 participants (mean age: 56.4 ± 14.3 years, 71% with hypertension) who underwent aldosterone–renin testing and echocardiography were included in the study. Data on demographics, clinical history, and medications, including calcium channel blockers and mineralocorticoid receptor antagonists (MRAs), were collected. We applied a generalized linear model (GLM) and a multivariable logistic regression model to estimate the relationships between ARR, PAC, and PRC with the risk of adverse LV remodeling and left ventricular hypertrophy (LVH) and further explored the dose–response relationship.

**Results:**

Of the 724 participants included in this study, GLM revealed that ARR was associated with greater left atrium size, left ventricular end-diastolic diameter, left ventricular mass, and left ventricular mass index. In adjusted multivariable regression analyses, one standard deviation (SD) of ARR emerged as a significant predictor of LVH occurrence [OR = 1.531 (95%CI, 1.041–2.251), *p* = 0.030], and compared with the first tertile of ARR, the third tertile of ARR had a 2.106-fold higher risk of LVH (*p*-trend <0.05), especially in participants without mineralocorticoid receptor antagonists (MRA). Furthermore, a significant dose–response relationship was observed between ARR and LVH risk (*p* overall <0.001, *p* non-linear = 0.079; *p* overall tests the overall association, while *p* non-linear tests for a non-linear trend between ARR and LVH risk).

**Conclusion:**

Elevated ARR is associated with an increased risk of adverse LV remodeling, and the presence of LVH may even occur at ARR levels below the clinical standard range, suggesting that ARR could serve as an early indicator of cardiac structural changes. Our results revealed that earlier targeted intervention with MRAs may be beneficial. However, this hypothesis requires confirmation in prospective and interventional studies, particularly those assessing the clinical and cost-effectiveness of early MR blockade. Our study provided a foundation for further exploration of this approach.

## 1 Introduction

Hypertension is a major preventable risk factor for cardiovascular disease (CVD) and premature death worldwide. Cardiovascular disease attributable to hypertension accounts for approximately 40% of all deaths in China (1, 2). Primary aldosteronism (PA) is the predominant cause of secondary hypertension, accounting for 5–13% in hypertensive patients (3, 4). It is characterized by excessive autonomous secretion of aldosterone by the adrenal glands, which resulted in overactivation of the mineralocorticoid receptor (MR) and then accelerated cardiovascular and renal damage through fibrosis, oxidative stress, and tissue remodeling (5, 6). Compared to patients with essential hypertension (EH), the increased risk of left ventricular hypertrophy (LVH), atrial fibrillation, and heart failure in those with PA was 2.29, 3.52, and 2.05, respectively (7). Of note, LVH, a precursor to cardiovascular disease, is always considered to occur earlier than other target organ damage (8). Building on this, recent studies have further reported that excess aldosterone can induce vascular damage independent of blood pressure, which may serve as an initiating factor for cardiac remodeling (9, 10). In addition to macrovascular damage, recent evidence has shown that excessive aldosterone secretion is also associated with significant microvascular impairment, suggesting a deleterious effect on microcirculation independent of blood pressure (11).

More studies supported that PA is a continuum of renin-independent aldosterone secretion affecting a broader population (12, 13), suggesting that the target organs might be impaired earlier than expected, especially with adverse left ventricular (LV) remodeling. The aldosterone-to-renin ratio (ARR) currently serves as the most effective screening indicator for PA; the majority of studies have only associated ARR with clinically overt PA (14, 15), often overlooking both non-PA populations and those with subclinical PA. Notably, this latter group, although lacking evident hypertension symptoms, has already started to accumulate abnormal aldosterone levels (8, 16). Expanding the application of ARR to include a wider population is thus vital. Such an expansion aids in the early detection and intervention of potential PA-related cardiovascular damage and provides a fresh perspective for evaluating cardiovascular disease risks.

To date, limited studies have focused on the early identification of adverse LV remodeling during the subclinical or pre-diagnostic stages of PA. Therefore, this study aimed to investigate the associations of the ARR, plasma aldosterone concentration (PAC), and plasma renin concentration (PRC) with LV remodeling and the occurrence of LVH in a population from Guangxi, China. We further explored whether these associations differ in individuals receiving or not receiving mineralocorticoid receptor antagonist (MRA) therapy.

## 2 Materials and methods

### 2.1 Study population

**Figure 1:**
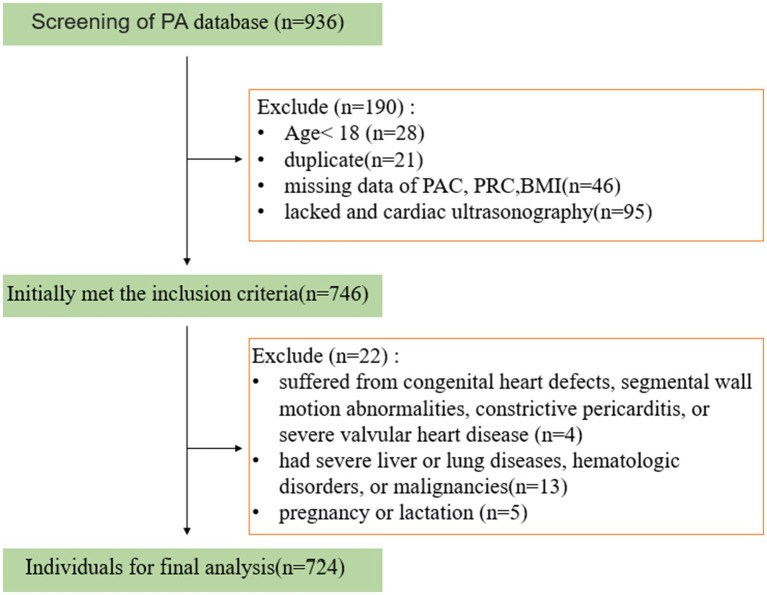
Flowchart illustrating the inclusion and exclusion criteria. PA, primary aldosteronism; PAC, plasma aldosterone concentration; PRC, plasma renin concentration; BMI, body mass index.

In this cross-sectional study, participants were consecutively recruited from hospitalized patients at the First Affiliated Hospital of Guangxi Medical University and the First People’s Hospital of Yulin City during September 2022–March 2024. The inclusion criteria were: all individuals who voluntarily joined the primary aldosteronism expanded screening program, except those meeting the following exclusion criteria. All participants completed PA screening, routine physical examination, biochemical detection, and the collection of demographic data, including lifestyle, disease history, and medication history. Medication use, including calcium channel blockers, beta-blockers, angiotensin-converting enzyme (ACE) inhibitors, angiotensin receptor blockers (ARBs), and MRAs, was recorded at baseline; the age of participants ranged from 18 to 89 years. Participants were excluded if they met any of the following criteria: 1. Age under 18; 2. Lacked PA screening and cardiac ultrasonography or key clinical data; 3. Suffered from congenital heart defects, segmental wall motion abnormalities, constrictive pericarditis, or severe valvular heart disease; 4. Had severe liver or lung diseases, hematologic disorders, or malignancies; 5. Were pregnant or nursing; and 6. Had known causes of secondary aldosteronism, including nephrotic syndrome, volume depletion, and inherited tubulopathies (e.g., Bartter or Gitelman syndromes). Finally, this study included a total of 724 participants for the subsequent analysis (Figure 1).

This study received ethical approval from the Ethics and Human Subject Committee of Guangxi Medical University, China (No. 2022-0193), and the Ethics and Human Subject Committee of the First Affiliated Hospital of Guangxi Medical University, China (No. 2023-K090-01). All participants provided written informed consent at the time of their recruitment.

### 2.2 Biochemical data index determination

The blood samples of participants were collected after remaining upright for at least 2 h and resting for 15 min. To minimize circadian influences on aldosterone and renin secretion, all blood samples were collected in the morning between 08:00 and 10:00. Before the assessment of aldosterone and renin levels, hypokalemia was corrected to ≥3.5 mmol/L. Patients on antihypertensive medication followed guidelines to discontinue dihydropyridine calcium channel blockers, β-blockers, ACE inhibitors, and ARBs, switching to non-dihydropyridine or α-blocker alternatives for 3 weeks to manage blood pressure (17). Blood samples were transported to the hospital laboratory within 2 h for testing aldosterone, renin, ARR, and electrolyte levels, following standard procedures. Aldosterone and renin measurements were performed using chemiluminescent microparticle immunoassays (CMIAs). The assays were conducted using the renin assay kit and aldosterone assay kit, both provided by AnTuo Bio. Blood pressure (BP) was measured in the morning using an Omron automatic sphygmomanometer. After participants had rested in a seated position for at least 15 min, two BP readings were taken at an interval of 30–60 s. If the systolic or diastolic BP differed by more than 10 mmHg between the two measurements, a third reading was taken. The average of the two or three readings was recorded. All procedures followed the Chinese Expert Consensus on Precision Medicine for Hypertension Diagnosis and Treatment (18).

### 2.3 Adverse LV remodeling parameters

Adverse LV remodeling parameters were assessed by professional sonographers using color Doppler echocardiography on a Philips EPIQ 7C system. Patients were positioned in the left lateral decubitus position and instructed to breathe calmly during the procedure. All measurements were taken at the end of expiration for at least three consecutive cardiac cycles, and the average value was taken as the final result. Left atrium size (LA), left ventricular end-diastolic diameter (LVDd), interventricular septum thickness (IVS), left ventricular posterior wall diameter (LVPWD), left ventricular mass (LVM), and left ventricular mass index (LVMI) were used as parameters for assessing adverse left ventricular remodeling.

LVMI was calculated as LVM/body surface area (BSA) (19). LVM = 0.8 × [1.04 × (LVDd + IVS + LVPWD)3 − (LVDd)3] + 0.6. BSA was determined using the Dubois formula: BSA (m^2^) = 0.007184 × height 0.725 × weight 0.425, where height is measured in centimeters (cm) and weight is measured in kilograms (kg).

LVH is defined as an LVMI greater than 95 g/m^2^ for females and greater than 115 g/m^2^ for males (20).

### 2.4 Medical history data collection

Comorbidities were ascertained based on documented clinical diagnoses and standardized diagnostic criteria. Hypertension was defined as a systolic blood pressure (SBP) ≥ 140 mmHg and/or a diastolic blood pressure (DBP) ≥ 90 mmHg in individuals not receiving antihypertensive therapy or a prior clinical diagnosis of hypertension with ongoing antihypertensive medication use. Individuals with newly diagnosed hypertension were also included. Type 2 diabetes mellitus (T2DM) was defined as a documented diagnosis, current use of glucose-lowering medications, or a fasting plasma glucose concentration ≥7.0 mmol/L. Atrial fibrillation (AF) was identified through medical history or confirmed by electrocardiographic findings. Heart failure (HF) was defined as a prior clinical diagnosis substantiated by typical symptoms, imaging evidence, or the use of heart failure–specific pharmacological treatment. Stroke history encompassed both ischemic and hemorrhagic events, as confirmed by clinical documentation.

### 2.5 Statistical analysis

To reduce skewness and make the data more suitable for statistical analysis, levels of PAC, PRC, and ARR were log-transformed when they were analyzed as continuous variables in subsequent analyses. Data were expressed as mean ± standard deviation (SD) for normally distributed variables, medians with interquartile range (IQR) for skewed variables, and number (percentages) for categorical variables. We further categorized PRC and ARR according to median values or clinical diagnostic criteria for group comparisons. Differences between skewed variables were tested using the Mann–Whitney *U* test; otherwise, normally distributed variables were tested using Student’s *t*-test, and categorical data were tested using the chi-square test. In addition, correlations between PAC, PRC, ARR, and echocardiographic parameters were estimated using Spearman’s analysis. Relationships between PAC, PRC, ARR, and adverse LV remodeling indicators, including left atrium diameter [LA], LVDd, IVS, LVPWd, LVM, and LVMI, were used by the generalized linear model (GLM). The dose–response relationship between ARR and LVH was explored using restricted cubic spline (RCS) analysis. Furthermore, we documented the relationship between ARR and LVH using a multivariable logistic regression model. The adjusted covariates in models contained sex, age, body mass index (BMI), serum potassium, diabetes, cardiovascular disease history, statin use, and categories of antihypertensive drugs (i.e., angiotensin-converting enzyme inhibitors, angiotensin II receptor blockers, beta-blockers, calcium channel blockers (CCBs), and MRAs). All statistical analyses were performed using R version 4.2.2 and SPSS 26.0, with a two-sided *p*-value of <0.05 considered statistically significant.

## 3 Results

### 3.1 Clinical characteristics of study population

Of the 724 participants, the average age was 56.4 ± 14.3 years old; the median levels of PAC, PRC, and ARR were 129.6 pg/mL,10.1 pg/mL, and 12.3, respectively; the majority of them were male (60.0%), of Han ethnicity (92%), and hypertensive patients (71.0%). When divided by the ARR threshold of 30, participants in the ARR ≥ 30 group were more likely to be female, older in age, and have higher systolic blood pressure, PAC, serum sodium, and chloride levels, but lower PRC and serum potassium and calcium levels (all *p* < 0.05). They also had a higher prevalence of diagnosed PA and hypertension and were more likely to be receiving MRAs, ARBs, and DHP CCBs than the ARR <30 group.

Additionally, the ARR ≥30 group had significantly increased cardiac structural parameters, including larger left atrial diameter, left ventricular end-diastolic diameter, interventricular septum thickness, posterior wall thickness, LV mass, and LVMI (all *p* < 0.05; Table 1).

**Table 1:** Baseline characteristics of the participants according to ARR.

Characteristics	Overall, *N* = 724	ARR<30, *N* = 529	ARR≥30, *N* = 195	*p*-value
Sex (*n*, %)				<0.001
Male	431 (60%)	351 (66%)	80 (41%)	
Female	293 (40%)	178 (34%)	115 (59%)	
Age (years)	56.4 ± 14.3	55.8 ± 14.8	58.2 ± 12.5	0.049
Ethnicity (*n*, %)				0.033
Han Chinese	668 (92%)	496 (94%)	172 (88%)	
Zhuang	52 (7.2%)	31 (5.9%)	21 (11%)	
Others	4 (0.6%)	2 (0.4%)	2 (1.0%)	
SBP (mmHg)	142.6 ± 24.6	141.3 ± 25.2	146.2 ± 22.7	0.012
DBP (mmHg)	86.7 ± 16.0	86.6 ± 16.8	87.0 ± 13.6	0.441
BMI (kg/m^2^)	24.2 ± 4.0	24.1 ± 4.2	24.4 ± 3.5	0.267
BSA (m^2^)	1.66 ± 0.19	1.66 ± 0.20	1.64 ± 0.18	0.1
PAC (pg/ml)	129.59 (94.13, 184.79)	123.68 (92.19, 175.94)	147.66(103.15, 219.65)	<0.001
PRC (pg/ml)	10.13 (3.87, 25.13)	16.72 (8.28, 33.03)	2.14 (1.00, 3.38)	<0.001
ARR	12.28 (5.53, 32.13)	8.13 (4.35, 14.38)	71.47 (40.41, 171.23)	\
K (mmol/l)	3.90 ± 0.51	3.92 ± 0.48	3.86 ± 0.56	0.029
Na (mmol/l)	139.7 ± 6.2	139.5 ± 3.6	140.2 ± 10.3	<0.001
Cl (mmol/l)	103.6 ± 4.2	103.1 ± 4.3	104.9 ± 3.8	<0.001
Ca (mmol/l)	2.26 ± 0.15	2.27 ± 0.14	2.24 ± 0.16	0.004
PA (*n*, %)	85 (12%)	15 (2.8%)	70 (36%)	<0.001
Hypertension (*n*, %)	515 (71%)	350 (66%)	165 (85%)	<0.001
T2DM (*n*, %)	285 (39%)	212 (40%)	73 (37%)	0.519
Stroke (*n*, %)	142 (20%)	101 (19%)	41 (21%)	0.561
AF (*n*, %)	10 (1.4%)	7 (1.3%)	3 (1.5%)	0.734
HF (*n*, %)	15 (2.1%)	13 (2.5%)	2 (1.0%)	0.377
OSAS (*n*, %)	47 (6.5%)	31 (5.9%)	16 (8.2%)	0.256
ACEI (*n*, %)	36 (6.5%)	27 (6.5%)	9 (6.5%)	0.985
ARB (*n*, %)	213 (38%)	149 (35%)	64 (45%)	0.031
BBs (*n*, %)	125 (22%)	97 (22%)	28 (19%)	0.456
DHP CCBs (*n*, %)	239 (39%)	158 (35%)	81 (49%)	0.001
MRA (*n*, %)	56 (10%)	28 (6.8%)	28 (21%)	<0.001
LD (*n*, %)	33 (6.1%)	25 (6.1%)	8 (5.9%)	0.942
Statins (*n*, %)	394 (72%)	305 (74%)	89 (66%)	0.057
AO (mm)	21.00 (20.00, 23.00)	21.00 (20.00, 23.00)	22.00 (20.00, 23.00)	0.262
LA (mm)	32.00 (28.50, 35.00)	31.00 (28.00, 35.00)	32.00 (29.00, 36.00)	0.017
PAD (mm)	23.00 (21.00, 24.00)	23.00 (21.00, 24.00)	23.00 (21.00, 24.00)	0.397
RV (mm)	20.00 (19.00, 22.00)	20.00 (19.00, 22.00)	20.00 (19.00, 22.00)	0.741
LVDd (mm)	45.00 (42.00, 48.00)	44.00 (41.00, 48.00)	46.00 (42.00, 49.00)	<0.001
IVS (mm)	10.50 (9.40, 12.00)	10.50 (9.30, 12.00)	10.80 (10.00, 12.00)	0.041
LVPWd (mm)	10.40 (9.20, 12.00)	10.30 (9.00, 11.80)	10.60 (9.50, 12.00)	0.044
LVM (g)	160.70(131.81, 200.70)	156.64(127.92, 196.5)	169.56(141.85, 213.90)	0.002
LVMI (g/m^2^)	98.16 (82.35, 119.59)	96.78 (80.63, 116.31)	105.96 (88.04, 125.77)	<0.001
EF (%)	21.00 (20.00, 23.00)	21.00 (20.00, 23.00)	22.00 (20.00, 23.00)	0.596
FS (%)	32.00 (28.50, 35.00)	31.00 (28.00, 35.00)	32.00 (29.00, 36.00)	0.420

Continuous variables were expressed as mean ± standard deviation (SD) for normally distributed data or as median with interquartile range (IQR, Q1–Q3) for non-normally distributed data. Categorical variables were presented as counts (percentages). The parameters were evaluated using univariate analyses. A paired *t*-test or Wilcoxon signed-rank test was used to compare variables. SBP, systolic blood pressure; DBP, diastolic blood pressure; BMI, body mass index; BSA, body surface area; PAC, plasma aldosterone concentration; PRC, plasma renin concentration; ARR, aldosterone-to-renin ratio; PA, primary aldosteronism; T2DM, Type 2 diabetes mellitus; AF, atrial fibrillation; HF, heart failure; OSAS, obstructive sleep apnea syndrome; ACEI, angiotensin-converting enzyme inhibitor; ARB, angiotensin II receptor blocker; BBS, beta-blockers; DHP CCBs: dihydropyridine calcium channel blockers; MRA, mineralocorticoid receptor antagonist; LD, loop diuretics; AO indicates aortic diameter; LA, left atrium diameter; PAD, pulmonary artery diameter; RV, right ventricle diameter; LVDd, left ventricular diastolic diameter; IVS, interventricular septal thickness; LVPWd, left ventricular posterior wall diameter; LVM, left ventricular mass; LVMI, left ventricular mass index; EF, ejection fraction; FS, fractional shortening.

As presented in Table 2, all echocardiographic parameters differed significantly between male and female participants. Notably, male participants had larger cardiac chamber dimensions [aortic diameter (AO), left atrium diameter (LA), pulmonary artery diameter (PAD), right ventricle diameter (RV), and left ventricular diastolic diameter (LVDd)], thicker walls (IVS and LVPWd), and greater LV mass and LVMI, while females had slightly higher ejection fraction (EF) and fractional shortening (FS).

**Table 2:** Comparison of echocardiographic parameters between male and female groups.

Characteristics	Overall, *N* = 724	Male, *N* = 431	Female, *N* = 293	*p*-value
AO (mm)	21.00 (20.00, 23.00)	22.00 (20.50, 24.00)	20.00 (19.00, 22.00)	<0.001
LA (mm)	32.00 (29.00, 35.00)	32.00 (30.00, 35.50)	30.00 (28.00, 34.00)	<0.001
PAD (mm)	23.00 (21.00, 24.00)	23.00 (21.00, 25.00)	22.00 (21.00, 24.00)	<0.001
RV (mm)	20.00 (19.00, 22.00)	21.00 (20.00, 22.00)	20.00 (18.00, 21.00)	<0.001
LVDd (mm)	45.00 (42.00, 48.00)	46.00 (43.00, 49.00)	43.00 (41.00, 46.00)	<0.001
IVS (mm)	10.50 (9.40, 12.00)	10.70 (9.60, 12.10)	10.20 (9.20, 11.60)	<0.001
LVPWd (mm)	10.40 (9.20, 12.00)	10.50 (9.50, 12.00)	10.00 (9.00, 11.50)	<0.001
LVM (g)	160.70 (131.81, 200.70)	174.74 (141.71, 214.23)	147.78 (123.30, 181.22)	<0.001
LVMI (g/m^2^)	98.21 (82.65, 119.59)	100.30 (83.18, 121.95)	95.50 (81.70, 116.13)	0.027
EF (%)	67.00 (63.00, 71.00)	66.00 (62.00, 70.00)	68.00 (63.00, 71.00)	0.012
FS (%)	37.00 (34.00, 40.00)	37.00 (34.00, 40.00)	38.00 (35.00, 40.00)	0.023

The Wilcoxon signed-rank test was used to compare variables. AO, aortic diameter; LA, left atrium diameter; PAD, pulmonary artery diameter; RV, right ventricle diameter; LVDd, left ventricular diastolic diameter; IVS, interventricular septal thickness; LVPWd, left ventricular posterior wall diameter; LVM, left ventricular mass; LVMI, left ventricular mass index; EF, ejection fraction; FS, fractional shortening.

**Figure 2:**
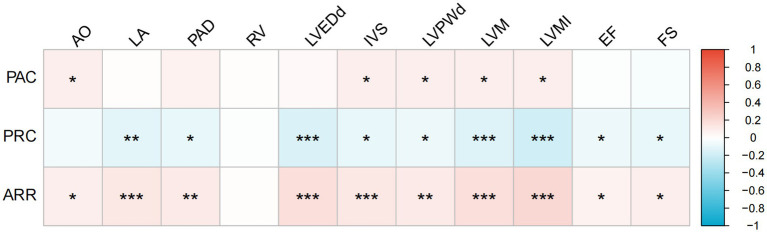
Heatmap of Spearman’s correlations between PAC, PRC, ARR, and echocardiographic parameters. Scheme 1 to red (positive correlation, *r* = 1). Asterisks denote significance levels: ‘*’ for *p* < 0.05, ‘**’ for *p* < 0.01, and ‘***’ for *p* < 0.001. AO, aortic root diameter; LA, left atrial size; PAD, pulmonary artery diameter; RV, right ventricular diameter; LVDd, left ventricular end-diastolic diameter; IVS, interventricular septal thickness; LVPWd, left ventricular posterior wall thickness; LVM, left ventricular mass; LVMI, left ventricular mass index; EF, ejection fraction; FS, fractional shortening; PAC, plasma aldosterone concentration; PRC, plasma renin concentration; ARR, aldosterone-to-renin ratio.

Spearman’s correlation analysis further indicated that PAC was positively correlated with aortic diameter (AO), IVS, LVPWd, LVM, and LVMI; PRC was negatively correlated with all the echocardiographic parameters, except for AO and right ventricle diameter (RV); and ARR was positively correlated with all the echocardiographic indicators, except for RV (Figure 2).

### 3.2 Associations of PAC, PRC, and ARR with adverse LV remodeling parameters

**Figure 3:**
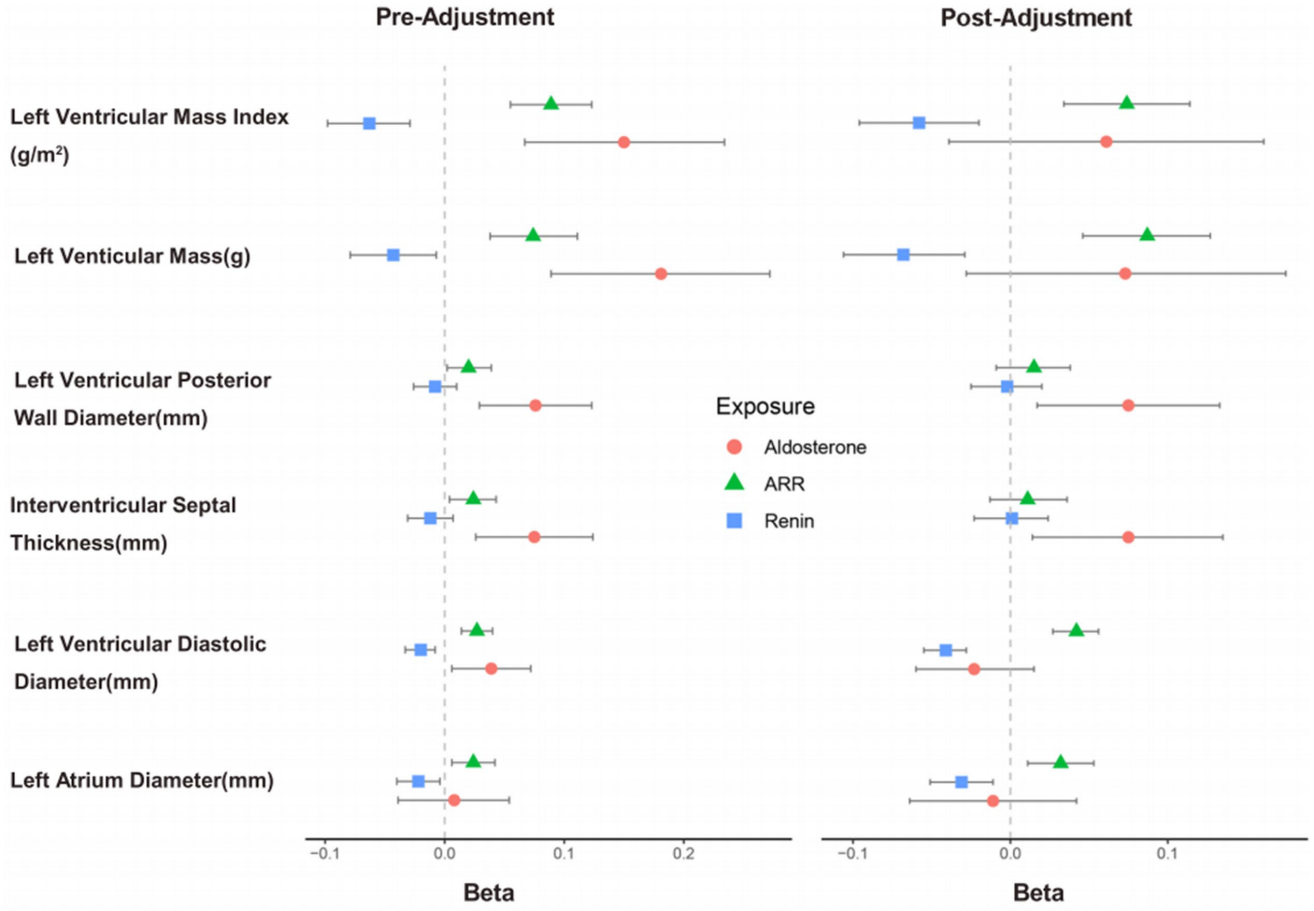
Generalized linear model analysis of the associations of aldosterone, renin, and ARR with adverse LV remodeling parameters. The correlations of aldosterone, renin, and ARR with left ventricular remodeling parameters, before and after adjusting for covariates. The right side presents the results after adjusting for sex, age, BMI, serum potassium, diabetes, cardiovascular disease history, statin use, and antihypertensive medication use. ARR indicates aldosterone-to-renin ratio.

GLM was used to assess the associations of PAC, PRC, and ARR with aLVR indicators, as illustrated in Figure 3. Prior to covariate adjustment, positive correlations were observed between PAC and ARR with LVDd, IVS, LVPWd, and LVMI. These correlations were statistically significant. After adjustment, the associations between PAC and LVM [*β* (95%CI), 0.073 (−0.028, 0.175), *p* = 0.156], LVDd [*β* (95%CI), −0.023 (−0.06, 0.015), *p* = 0.232], and LVMI [*β* (95%CI), 0.061 (−0.039, 0.161), *p* = 0.232] were not significant. Conversely, ARR consistently showed significant positive correlations with LVDd [*β* (95%CI), 0.042 (0.027, 0.056), *p* < 0.001], LVM [*β* (95%CI), 0.087 (0.046, 0.127), *p* < 0.001], and LVMI [*β* (95%CI), 0.074 (0.034, 0.114), *p* < 0.001].

### 3.3 Higher ARR is associated with increased LVH risk

**Figure 4:**
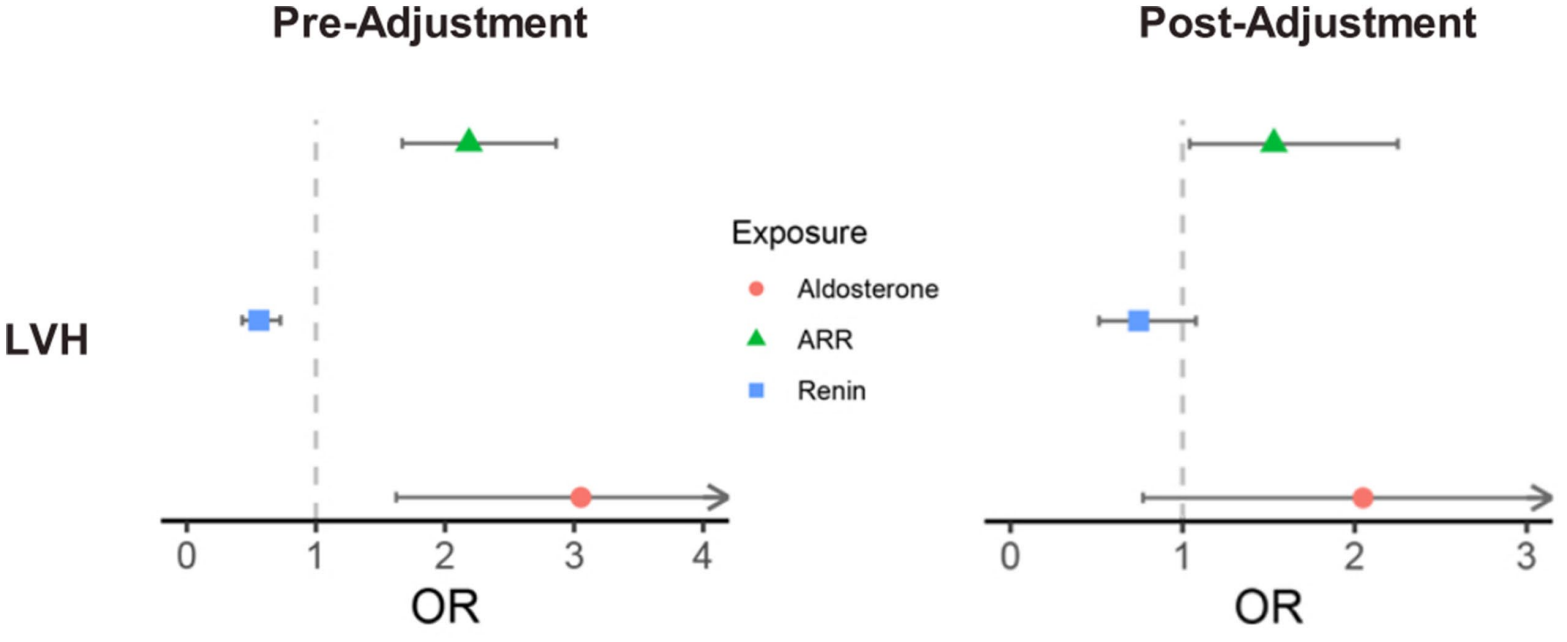
Association between aldosterone, renin, ARR, and LVH risk. The left side shows the results before adjusting for covariates, while the right side presents the results after adjusting for sex, age, BMI, serum potassium, diabetes, cardiovascular disease history, statin use, and antihypertensive medication use. LVH, left ventricular hypertrophy; ARR, aldosterone-to-renin ratio.

Before adjustment of potential covariates, a binary logistic regression analysis showed that PAC [OR (95% CI), 3.053 (1.622, 5.747), *p* = 0.001] and ARR [OR (95%CI), 2.186 (1.669, 2.861), *p* < 0.001] were positively correlated with the presence of LVH (defined as LVMI >115 g/m^2^ in males and >95 g/m^2^ in females), whereas PRC [OR (95%CI), 0.558 (0.429, 0.725), *p* < 0.01] was inversely related to LVH. After adjusting for potential covariates, only ARR remained significantly associated with the presence of LVH [OR (95% CI), 1.531 (1.041–2.251), *p* = 0.03] (Figure 4). Specifically, participants in the third tertile of ARR had 2.106-fold higher odds of LVH than those in the first tertile, and this trend was statistically significant (*p*-trend <0.05) (Table 3).

**Table 3:** Associations between ARR and LVH.

LVH	T1		T2		T3		*p* for trend
	OR (95%CI)	*p*-value	OR (95%CI)	*p*-value	OR (95%CI)	*p*-value	
Model 1	Reference	–	2.493 (1.665, 3.735)	<0.001	3.262 (2.182, 4.876)	<0.001	<0.001
Model 2	Reference	–	1.890 (1.213, 2.946)	0.005	2.106 (1.339, 3.312)	0.001	0.003

The tertiles of the ARR are designated as T1, T2, and T3, respectively; Mode 1: unadjusted for covariate; Mode 2: adjusted for covariates such as sex, age, body mass index (BMI), serum potassium, diabetes, cardiovascular disease history, statin use, and categories of antihypertensive drugs. ARR, aldosterone-to-renin ratio; LVH, left ventricular hypertrophy.

**Figure 5:**
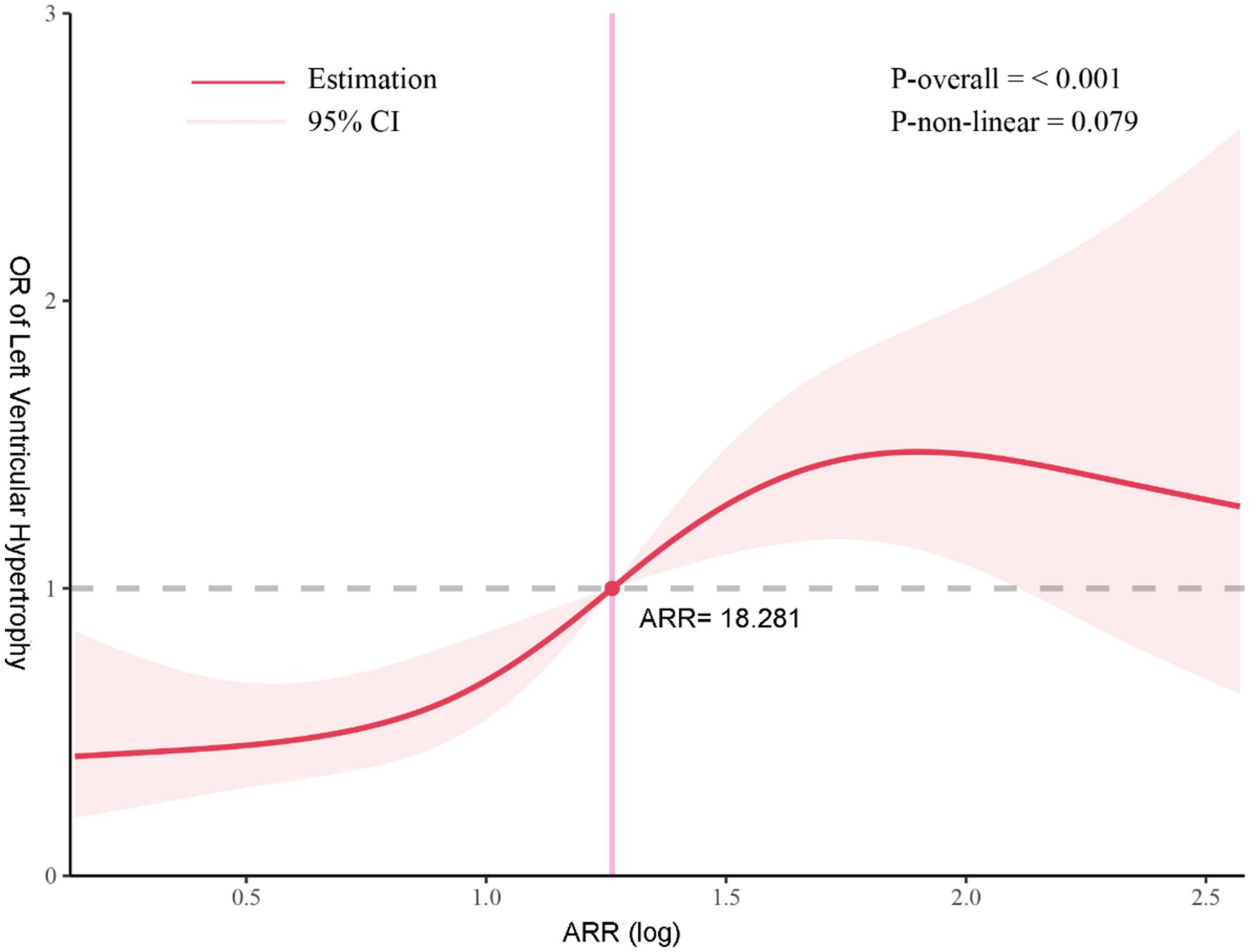
Assessment of the association between ARR and LVH risk. Association between ARR and LVH risk after excluding the 0.5th percentile extreme values from all participants. The solid line represents the estimated values, and the red shaded area indicates the 95% confidence interval. The vertical axis shows the OR of left ventricular hypertrophy, while the horizontal axis represents aldosterone-to-renin ratio values. *p*-overall and *p*-non-linear indicate the statistical significance of the overall correlation and the non-linear relationship, respectively. The intersection of the red vertical line and the gray horizontal dashed line marks the log-ARR value at which the OR equals 1.

Further RCS analysis showed a significant positive association between ARR and the risk of LVH (*p* overall <0.001), and the test for non-linearity was not statistically significant (*p* = 0.079), suggesting an approximately linear relationship (see Figure 5).

### 3.4 Effect of ARR on LVH risk in MRA medication subgroup

In addition, we further explored the associations between ARR and the presence of LVH on the condition of MRA medication status (Table 4). The findings indicated that higher ARR was associated with greater odds of LVH [OR (95%CI), 1.669 (1.217, 2.289), *p* = 0.001, Model 2] in the non-MRA group, and the association remained significant even after adjustments for SBP [OR (95%CI), 1.692 (1.222, 2.342), *p* = 0.002] or DBP [OR (95% CI), 1.706 (1.239, 2.349), *p* = 0.001]. Nevertheless, no significant relationship was detected between ARR and LVH risk in the MRA-treated group (*p* > 0.05). The interaction between ARR and MRA usage was not statistically significant (*p*-interaction >0.05).

**Table 4:** Relationship between ARR and LVH risk based on the usage of MRA medication.

LVH	Non-MRA (*n* = 450)		MRA group (*n* = 51)		*p*-interaction
	OR (95%CI)	*p*-value	OR (95%CI)	*p*-value	
Model 1	2.235 (1.676, 2.980)	<0.001	1.860 (0.828, 4.174)	0.133	0.675
Model 2	1.669 (1.217, 2.289)	0.001	3.468 (0.822, 14.641)	0.091	0.789
Model 3	1.692 (1.222, 2.342)	0.002	1.235 (0.236, 6.466)	0.803	0.742
Model 4	1.706 (1.239, 2.349)	0.001	1.428 (0.294, 6.931)	0.658	0.664

To analyze the impact of MRA medication on the predictive value of the aldosterone-to-renin ratio (ARR) for LVH risk, participants were categorized into two groups based on MRA usage. Four binary logistic regression models were established. Model 1 included no covariate adjustments. Model 2 was adjusted for sex, age, BMI, serum potassium levels, diabetes, history of cardiovascular disease, and statin use. Model 3 added an adjustment for systolic blood pressure, and Model 4 included an additional adjustment for diastolic blood pressure. MRA, mineralocorticoid receptor antagonist; LVH left ventricular hypertrophy.

## 4 Discussion

Our study demonstrated that higher ARR but not PAC or PRC was positively associated with the risk of LVH, even when below conventional PA screening thresholds. These findings align with recent studies indicating that ARR can forecast cardiac metabolic disturbances in non-PA populations (21). Only ARR emerged as a significant predictor of LVH in the adjusted binary logistic regression analysis. Moreover, the analysis indicated that LVH risk increases at ARR levels lower than common PA screening thresholds, highlighting ARR’s potential as a biomarker for the early detection of LVH. Notably, the association between ARR and LVH was absent in patients receiving MRA treatment, suggesting that MRA may attenuate aldosterone-related cardiac damage—a key finding that warrants further exploration.

Rossi et al. (22) first reported that PA patients with adrenalectomy significantly mitigated adverse LV remodeling compared to those with EH, and the association between PA and adverse LV remodeling was supported by subsequent research (23, 24). Several subsequent studies confirmed that PA-related cardiac damage, including LVH and myocardial fibrosis, is not fully explained by blood pressure elevation alone (7, 25). Possible explanations stem from MR being extensively expressed in tissues, and oxidative stress, fibrosis, tissue remodeling, and inflammation could occur when MR is excessive activated (26, 27). Our study is consistent with this body of evidence but further refines it by showing that among PAC, PRC, and ARR, only ARR was significantly associated with LVH after adjustment for confounding factors, including blood pressure. This is in line with a retrospective Japanese study, which found that baseline PAC did not significantly correlate with LVMI, whereas PAC levels of post-captopril or saline infusion test had significantly correlated with LVMI (28). This supports the notion that ARR, by reflecting both aldosterone and renin suppression, is a more robust index of autonomous aldosterone activity and its cardiovascular impact. Moreover, the review by Doumas et al. (29) emphasizes the significant and practical clinical value of ARR measurement in patients with resistant hypertension, as it facilitates the identification of primary aldosteronism—the most common cause of secondary hypertension. They advocate for the broader application of ARR testing in clinical practice to uncover subclinical or atypical presentations of PA. Our findings support and extend this viewpoint by indicating that ARR may serve as an early cardiovascular risk indicator, even in the absence of overt PA.

Our data indicate that individuals with suppressed renin activity are more likely to exhibit LVH, which may reflect heightened MR activation. This observation is consistent with previous studies suggesting that low-renin states often signal more pronounced aldosterone excess and target organ damage (30, 31). The composite index ARR, combining plasma aldosterone and renin levels, therefore offers a more integrated reflection of MR activity and demonstrates superior predictive value for adverse cardiac remodeling compared to aldosterone or renin alone.

PA leads to autonomous secretion of aldosterone, which impacts adverse LV remodeling and LVH through two pathways: indirectly by increasing blood pressure and directly by affecting non-epithelial myocardial cells (32). If heart structural changes exacerbate in a vicious cycle, they can further compromise left ventricular contractility, leading to reduced ejection fraction and ultimately heart failure (33). The primary cause of this cardiovascular damage is excessive MR activation. Blocking MR activation through adrenalectomy or regular MRA administration can slow disease progression and even reverse LVH (23).

In our analysis, we found that, in contrast to patients not taking MRAs, no statistically significant association was observed between ARR and LVH in the MRA group. Although this lack of association might be due to the small number of patients in this subgroup, the observed trend could provide some insights into the role of MRA treatment in modifying the effect of ARR on LVH. Further studies with larger sample sizes would be needed to substantiate these findings. Notably, after adjusting for SBP and DBP, the predictive effect of ARR on LVH remained consistent across all models, indicating that the protective effect of MRA is not primarily achieved through blood pressure reduction. These findings support that the benefits of MRA treatment for PA patients extend beyond blood pressure control, primarily by inhibiting excessive MR activation, thus potentially reducing aldosterone’s direct damage to the heart. A study found that spironolactone could reverse LVH in PA patients, but no linear relationship was observed between blood pressure changes and LVMI changes (34), which aligns with our findings. Additionally, Hundemer et al. (30) found that PA patients with persistently suppressed renin activity faced additional cardiovascular risks, but this risk did not increase when renin was not suppressed, further highlighting the significant role of autonomous aldosterone secretion and MR activation in cardiovascular events. Collectively, these studies highlight the exploratory nature of the findings, suggesting that the most significant benefit of MRA treatment, beyond blood pressure control, is likely its ability to mitigate the direct cardiac damage caused by aldosterone. However, the limited sample size in our study warrants careful interpretation of these results. We acknowledge that further research, particularly involving larger cohorts and randomized controlled trials (RCTs), is necessary to fully elucidate the spectrum of MRA effects on cardiovascular remodeling and their clinical implications.

A notable finding from our study is that the risk of developing LVH begins to significantly increase even when ARR levels are below the commonly accepted thresholds for PA screening, and this risk escalates as ARR increases, suggesting that MR-mediated cardiovascular damage commences prior to a PA diagnosis (35). Our findings once again challenge the concept of PA as a binary disease, aligning with prior research that suggests PA severity progressively intensifies from mild to severe (12, 36, 37). Furthermore, these results highlight the potential value of ARR in predicting LVH across a broader population. It is well established that PA accelerates cardiovascular disease progression (7, 38). While MRAs can control disease progression and protect target organs, current guidelines primarily recommend ARR testing for patients with clinical symptoms (39). Our study indicates that elevated ARR is associated with significant cardiovascular risk not only in PA patients but also in a broader population. RCS analysis demonstrated a positive and approximately linear relationship between ARR and LVH risk. These findings raise the possibility that cardiovascular harm may accumulate gradually along the continuum of aldosterone excess. Accordingly, earlier consideration of MRA therapy for individuals with elevated ARR—even in the absence of confirmed PA—may warrant further investigation. Broader ARR screening and prospective studies are needed to determine whether early intervention could effectively delay disease progression and reduce cardiac remodeling.

The strengths and novelty of our study include a thorough analysis of ARR, PAC, PRC, echocardiography, biochemical data, and medical histories of all participants. Furthermore, our study design conceptualizes PA as a spectrum of diseases, eschewing a binary classification and emphasizing the examination of potential subclinical PA and the normotensive population. However, our study has several limitations. First, as an observational study, it is susceptible to both unpredictable confounding factors and residual confounding from unmeasured variables, such as dietary sodium intake or out-of-clinic blood pressure, which were not accounted for in our analysis. Second, this two-center study was confined to Guangxi, China, within a predominantly single-ethnicity (Han Chinese) population, which may limit the generalizability of our findings to other regions and care settings. Moreover, due to the inherent limitations of a cross-sectional study, causal relationships between ARR and LV remodeling cannot be established. However, our results suggested a strong association between ARR and LV remodeling. Finally, the measurements of ARR, PAC, and PRC were subject to individual variability and were not repeated multiple times to ensure greater accuracy. Additionally, since our study focused on the initial screening for PA (e.g., using ARR), a comprehensive assessment for other causes of secondary hypertension was not performed. As a result, the prevalence of other secondary hypertension etiologies may have been underestimated. In future research, we will conduct a prospective multicenter cohort with repeated ARR/PAC/PRC measurements, richer covariate collection, confirmatory testing for PA, and longitudinal echocardiography to clarify temporality and enhance generalizability.

## 5 Conclusion

This cross-sectional study conducted in Guangxi has revealed the potential efficacy of the elevation of ARR as a predictor for adverse LV remodeling, demonstrating significant associations with cardiovascular disease risk even at levels below current screening thresholds for PA.

## Data Availability

The raw data supporting the conclusions of this article will be made available by the authors, without undue reservation.
